# Water extract from artichoke ameliorates high-fat diet-induced non-alcoholic fatty liver disease in rats

**DOI:** 10.1186/s12906-022-03794-9

**Published:** 2022-11-24

**Authors:** Aihua Deng, Fengying Liu, Xuchong Tang, Yun Wang, Peng Xie, Qifu Yang, Bing Xiao

**Affiliations:** 1 College of Life and Environmental Science, Hunan University of Arts and Science, Changde, 415000 China; 2 College of Chemical Engineering, Huaqiao University, Xiamen, 361021 China; 3 Institute for Developmental and Regenerative Cardiovascular Medicine, Xinhua Hospital, School of Medicine, Shanghai Jiao Tong University, Shanghai, 200092 China

**Keywords:** Artichoke, Non-alcoholic fatty liver disease, High-fat diet, Antioxidant, Insulin resistance

## Abstract

**Background:**

The “multiple-hit” hypothesis is currently the most widely accepted theory for non-alcoholic fatty liver disease (NAFLD) pathogenesis. The present study aimed to investigate the effects of the water extract of artichoke (WEA) on NAFLD and its underlying mechanism.

**Methods:**

Rats were fed a high-fat diet (HFD) for 8 weeks to induce NAFLD and then treated with WEA at three doses (0.4, 0.8, and 1.6 g/kg body weight, BW) for 8 weeks. At the end of the intervention, serum biochemical parameters, hepatic antioxidant capacity, hepatic levels of pro-inflammatory cytokines, liver histopathology, hepatic inflammatory gene and lipid metabolism gene expression, and Akt and p-Akt (S473) protein levels were determined.

**Results:**

The body weight, liver weight, liver triglyceride (TG) and serum levels of TG, total cholesterol, low-density lipoprotein cholesterol, alanine aminotransferase, aspartate aminotransferase, glucose, and insulin were all significantly reduced in the WEA-treated groups (0.8 and 1.6 g/kg BW) compared with the HFD group (*P* < 0.01). A significant decrease in hepatic content of malondialdehyde (*P* < 0.01) and glutathione (*P* < 0.01), as well as a significant increase in liver superoxide dismutase activity (*P* < 0.01) were observed in WEA-treated groups (0.8 and 1.6 g/kg BW) compared to the HFD group. In addition, there was a marked decrease in the hepatic levels of pro-inflammatory cytokines (TNF-α, IL-1β, and IL-6) in the WEA-treated groups compared to the HFD group (*P* < 0.01). In line with these findings, the histopathology of the livers of rats treated with WEA (0.8 and 1.6 g/kg BW) showed a decrease in steatosis, ballooning, and lobular inflammation. Mechanistically, the reduced hepatic TG content might be related to the downregulation of lipogenic genes (SREBP1c, FASN, SCD1) and upregulation of lipolytic gene (PPARα), and the improved insulin signaling might be associated with the observed increase in antioxidant activity and reduction in inflammation in the WEA-treated groups.

**Conclusion:**

The hepatoprotective role of WEA in NAFLD may be attributed to its anti-steatotic, antioxidant, anti-inflammatory, and anti-insulin resistance effects.

**Supplementary Information:**

The online version contains supplementary material available at 10.1186/s12906-022-03794-9.

## Introduction

Non-alcoholic fatty liver disease (NAFLD), a condition characterized by hepatic lipid accumulation in the absence of excess alcohol intake, is the most common form of chronic liver disease worldwide. With time, it may progress to non-alcoholic steatohepatitis (NASH), advanced fibrosis, cirrhosis, and ultimately hepatocellular carcinoma [1]. NAFLD is categorized as a “multiple-hit” chronic liver disease associated with disorders including metabolic dysfunction, steatosis, oxidative stress, inflammation, and insulin resistance. The aforementioned are believed to be important causative factors contributing to its development and progression, and thus provide a more accurate explanation of the pathogenesis of NAFLD [2]. Accordingly, the ideal drug candidate for NAFLD should improve steatosis, hepatic inflammation, and oxidative stress, while ameliorating glucose metabolism, insulin resistance, and obesity, as weight loss has been shown to improve the histological characteristics of NAFLD [3]. However, there are currently no approved drugs for the treatment of NAFLD. Although metformin, statins, and fibrates have been tested as NAFLD treatment options in clinical trials, these drugs have significant adverse effects, such as the risk of infection and osteoporosis [4]. Owing to this, novel treatment candidates with high efficacy and minimal side effects are urgently required for NAFLD therapy.

Currently, the effects of herbal extracts and natural products on NAFLD have received increasing attention, and a number of these studies have reported on numerous herbal products with potent effects against NAFLD [5, 6]. Artichoke (*Cynarascolymus* L.), a member of the Asteraceae family, is widely used as a healthy food and popular traditional herbal medicine for hepatoprotection [7–9]. Artichokes contain abundant polyphenols (especially chlorogenic acid and cynarin), flavonoids, and their derivatives, and have thus attracted widespread attention because of their multiple pharmacological functions, including anti-oxidative, hypolipidemic, anti-inflammatory, and anticancer effects [10–12]. For example, artichoke leaf extract has been reported to attenuate oxidative stress in rats fed a high-cholesterol diet [13] and streptozotocin-induced diabetic rats [14]. A recent clinical study demonstrated that artichoke leaf extract decreased plasma levels of aspartate aminotransaminase (AST) and alanine aminotransferase (ALT) in patients with non-alcoholic steatohepatitis (NASH) [9]. Similarly, in our previous work, we found that the ethanol extract of artichoke exhibited significant protective effects against acute alcohol-induced liver injury, possibly through anti-inflammatory and antioxidant effects [15].

Herein, through a novel extraction process, we obtained a water extract of artichoke (WEA) containing much higher contents of chlorogenic acid and cynarin than a previously reported artichoke ethanol extract [15]. Further, we investigated the effects and mechanism of the WEA on NAFLD induced by a high-fat diet (HFD) in rats. The effects of WEA administration on body weight, liver weight, liver triglycerides (TG), serum biochemical parameters, hepatic antioxidant capacity, hepatic levels of pro-inflammatory cytokines, and liver histopathology were determined. The mechanisms of the hepatoprotective effects of WEA were deciphered by quantifying the markers of oxidative stress, inflammatory cytokines, insulin signaling, and hepatic gene expression of lipid metabolism.

## Materials and methods

### Materials

The following biochemical kits were purchased from Nanjing Jiancheng Institute of Biotechnology (Nanjing, China) and used to estimate the corresponding biomarkers: TG (cat. no. A110–1-1); total cholesterol (TC) (cat. no. A111–1-1); low-density lipoprotein cholesterol (LDL-C) (cat. no. A113–1-1); high-density lipoprotein cholesterol (HDL-C) (cat. no. A112–1-1); AST (cat. no. C010–2-1); ALT (cat. no. C009–2-1); glucose (GLU) (cat. no. A154–1-1); insulin (cat. no. H203–1-1); malondialdehyde (MDA) (cat. no. A003–1-2); glutathione (GSH) (cat. no. A006–2-1); superoxide dismutase (SOD) activity (cat. no. A001–1-2). Enzyme-linked immunosorbent assay (ELISA) kits for tumor necrosis factor-alpha (TNF-α) (cat. no. ml002859), interleukin-1 beta (IL-1β) (cat. no. ml037361), and interleukin-6 (IL-6) (cat. no. ml064292) were purchased from Enzyme-Linked Biotechnology (Shanghai, China). Reagents and kits used for RNA extraction (cat. no. 9767), reverse transcription (cat. no. RR037A), and real-time polymerase chain reaction (PCR) (cat. no. RR430A) were purchased from Takara Biotechnology (Dalian, China). Primers were purchased from BioSune Biotechnology (Shanghai, China). Akt (cat. no. sc-5298) and p-Akt (S473) (cat. no. sc-293,125) antibodies were purchased from Santa Cruz Biotechnology (Shanghai, China). All other chemicals used in this study were of analytical grade.

### Preparation of WEA

WEA was supplied by Huimei Agricultural Science and Technology Co. Ltd. (Hunan, China). Briefly, the extraction process was as follows: fresh artichoke was milled, followed by extraction with hot water (1:5, w/w) at 90 °C for 1.5 hours. The filtered extract was then concentrated by rotary evaporation. Next, a spray-drying method was used to obtain a water extract of the artichoke product. The artichoke extract product was quantified using a high-performance liquid chromatography (HPLC) system (Santa Clara, CA, USA). Briefly, the mobile phase was composed of 0.2% acetonitrile and phosphomolybdic acid in an aqueous solution. The concentration of acetonitrile was gradually increased from 5 to 20% in 10 min and then increased from 20 to 30% in 15 min. Next, the concentration of acetonitrile was gradually decreased from 30 to 5% in 25 min and then maintained at 5% for 30 min. The flow rate was set at 1.0 mL/min and the column temperature was 30 °C. The detection wavelength was set at 330 nm.

### Animals

Male Sprague Dawley (SD) rats (8-weeks-old) (𝑛 = 40) (certificate No. SCXK (Hu)2012–0002) weighing 220 ± 5 g were purchased from the Shanghai Laboratory Animal Center (Shanghai, China) and used for experimental procedures after 7 days of acclimation. All animals were maintained under controlled conditions (22 ± 2 °C, 60 ± 5% humidity, and 12–12 h light–dark cycle), and were given free access to standard laboratory animal feed and water. A normal chow diet (fat contributed 10% calories) and HFD (fat contributed 45% calories) were purchased from Jiangsu Medicine Biological & Pharmaceutical Company (Yangzhou, China). All animal procedures were approved by the Institutional Animal Care and Use Committee at Huaqiao University (Approval No. HQ-ECLA-20200316).

### Experimental method

The male SD rats were randomly assigned to five groups (*n* = 8 per group): chow diet control, HFD, HFD + WEA 0.4 (WEA 0.4 g/kg body weight, BW), HFD + WEA 0.8 (WEA 0.8 g/kg BW), and HFD + WEA 1.6 (WEA 1.6 g/kg BW). Rats were fed a HFD for 8 weeks to induce NAFLD and then treated with WEA for 8 weeks. Rats in the WEA treatment groups received WEA at doses of 0.4, 0.8, and 1.6 g per kg BW daily via oral administration. The chow diet and HFD groups were gavaged with an equal volume of saline solution (0.9% w/v). After intervention, the body weights of the rats were measured, and the animals were fasted overnight. Following this, blood samples were obtained from the hearts by cardiac puncture, and serum samples were preserved at − 80 °C for biochemical analyses. Liver weights were measured, and liver samples isolated after perfusion with PBS were processed for biochemical and histopathological studies, RNA extraction, and western blotting analysis.

### Liver index calculation

Liver index, an indicator of hepatic steatosis, was calculated using the following formula: liver index = [liver weight (g)/body weight (g)] × 100 [16].

### Determination of biochemical parameters

Serum TG, TC, LDL-C, HDL-C, AST, ALT, GLU, and insulin levels were determined using commercially available kits following the manufacturer’s instructions. Lysis of rat liver tissue was performed as previously described [15], with minor modifications. Briefly, liver tissue (~ 100 mg) was cut into slices and then homogenized with saline solution (0.9% w/v) in an ice bath. The samples were centrifuged at 12,000 rpm for 10 min at 4 °C, and the supernatant was collected and stored at − 80 °C until analysis. Liver MDA and GSH levels, SOD activity, and TG content were determined using commercial kits according to the manufacturer’s instructions.

### Histopathological analysis

Liver histopathological changes were assessed as previously described [17]. Briefly, 200 mg tissue sections were removed from the same region of the right lobe of the liver from animals in each group, fixed in 10% neutral formalin, embedded in paraffin, and then 5 μm sections were cut and stained with hematoxylin and eosin (H&E). Histopathological analysis was performed, and quantitative scoring of the morphological data was evaluated by an expert blinded to the analysis of hepatic steatosis. Hepatic fat accumulation was observed by three examiners independently using a light microscope and scored as follows: steatosis (0, < 5%; 1, 6–33%; 2, 34–66%; 3, > 66%), ballooning (0, 0 foci; 1, < 2 foci; 2, 2–4 foci; 3, > 4 foci, per 200× field), and inflammation (0, 0 foci; 1, < 2 foci; 2, 2–4 foci; 3, > 4 foci, per 200× field).

### RNA extraction and real-time PCR analysis

Total RNA was extracted using TRIzol reagent (Invitrogen, Carlsbad, CA, USA), following the manufacturer’s protocol. Real-time PCR was performed as previously described [18]. Briefly, cDNA was synthesized from 1 μg of RNA using a primer mix (oligo-dT/random primers) and Superscript reverse transcriptase (Takara, Dalian, China), according to the manufacturer’s instructions. Real-time PCR was performed in triplicate for each sample, using a 20 μL reaction volume and a SYBR Green universal PCR mix (Takara). The thermal cycling conditions were as follows: 95 °C for 3 min (initial denaturation), 95 °C for 10 s repeated in 40 cycles (denaturation), and 60 °C for 30 s (annealing/extension). The relative expression of the target genes was normalized to the expression of GAPDH, and the fold change was calculated using the 2^−ΔΔCt^ method. The primer sequences used for the real-time PCR are listed in Table 1.

**Table 1:** Primer Sequences for real-time PCR

Gene	Primer sequences (5′ > 3′)
SREBP1c-L	TTTGATGCCCCCTATGCTGG
SREBP1c-R	GCCCAGAGAAGCAGGAGAAG
SCD1-L	CCAAGCTGGAGTACGTCTGG
SCD1-R	AGACCTTGGAGGAGGGGATC
FASN-L	AAACACTGGTGTCTGGGTGG
FASN-R	CACCATGCTGTAGCCCAGAA
PPARα-L	TCCCGTTCACAAGAGCTGAC
PPARα-R	GGACGCAGGCTCTACTTTGA
TNFα-L	TCGTCTACTCCTCAGAGCCC
TNFα-R	AATTCTGAGCCCGGAGTTGG
IL-1β-L	AGCTTCAGGAAGGCAGTGTC
IL-1β-R	TCCTCATCCTGGAAGCTCCA
IL-6-L	ACTTCACAAGTCCGGAGAGG
IL-6-R	CCTCCGACTTGTGAAGTGGT
GAPDH-L	GAAGCTGGTCATCAACGGGA
GAPDH-R	ACGACATACTCAGCACCAGC

*GAPDH* glyceraldehyde 3-phosphate dehydrogenase

### Western blotting

Western blotting was performed as previously described [18]. Briefly, liver tissue proteins were extracted using a lysis buffer containing protease or phosphatase inhibitors. Protein concentrations were determined using a BCA protein assay kit (cat. no. 23225, Thermo Scientific, USA). Protein samples were separated using 10% SDS-PAGE and transferred to a PVDF membrane (cat. no. IPVH00010, Millipore, USA). Western blotting was performed using antibodies against Akt (cat. no. sc-5298; Santa Cruz Biotechnology, USA), phospho-Akt (Ser473) (cat. no. sc-514,032; Santa Cruz Biotechnology, USA), and β-actin (cat. no. 4967; Cell Signaling Technology, USA). Specific secondary antibodies labeled with horseradish peroxidase (HRP; 1:5000 dilution; cat. no. ab205719, Abcam, USA) and an enhanced chemiluminescence (ECL) detection kit (cat. no. 32106, Thermo Scientific, USA) were used to detect protein signals. Protein band intensities were quantified using ImageJ software (ver. 1.44p; National Institutes of Health, Bethesda, MD, USA).

### Statistical analysis

Data are presented as mean ± standard deviation (SD). Statistical analysis was performed by one-way analysis of variance (ANOVA) followed by post-hoc Tukey’s test using GraphPad Prism software (version 6.0; GraphPad Software, Inc., California, USA). Statistical significance was set at *P* < 0.05.

## Results

### Quantification of WEA

**Fig. 1:**
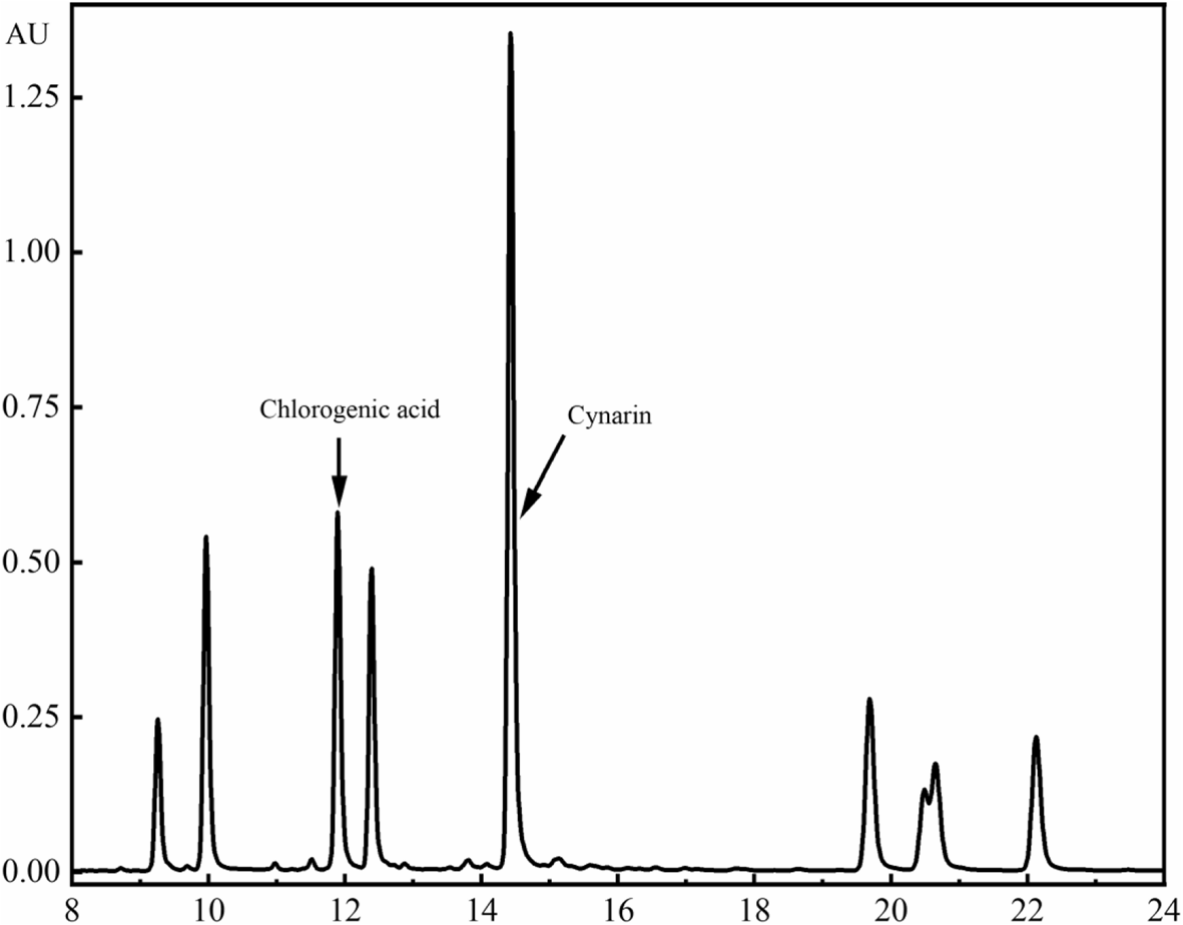
Characteristic and quantification analysis of chlorogenic acid and cynarin in WEA by high performance liquid chromatography at 330 nm

The artichoke extract product was quantified using a HPLC system. As showed in Fig. 1, The maximum elution times for chlorogenic acid (1.2%) and cynarin (4.8%) were 11.893 and 14.426 min, respectively.

### Effect of WEA on body and liver weights

**Fig. 2:**
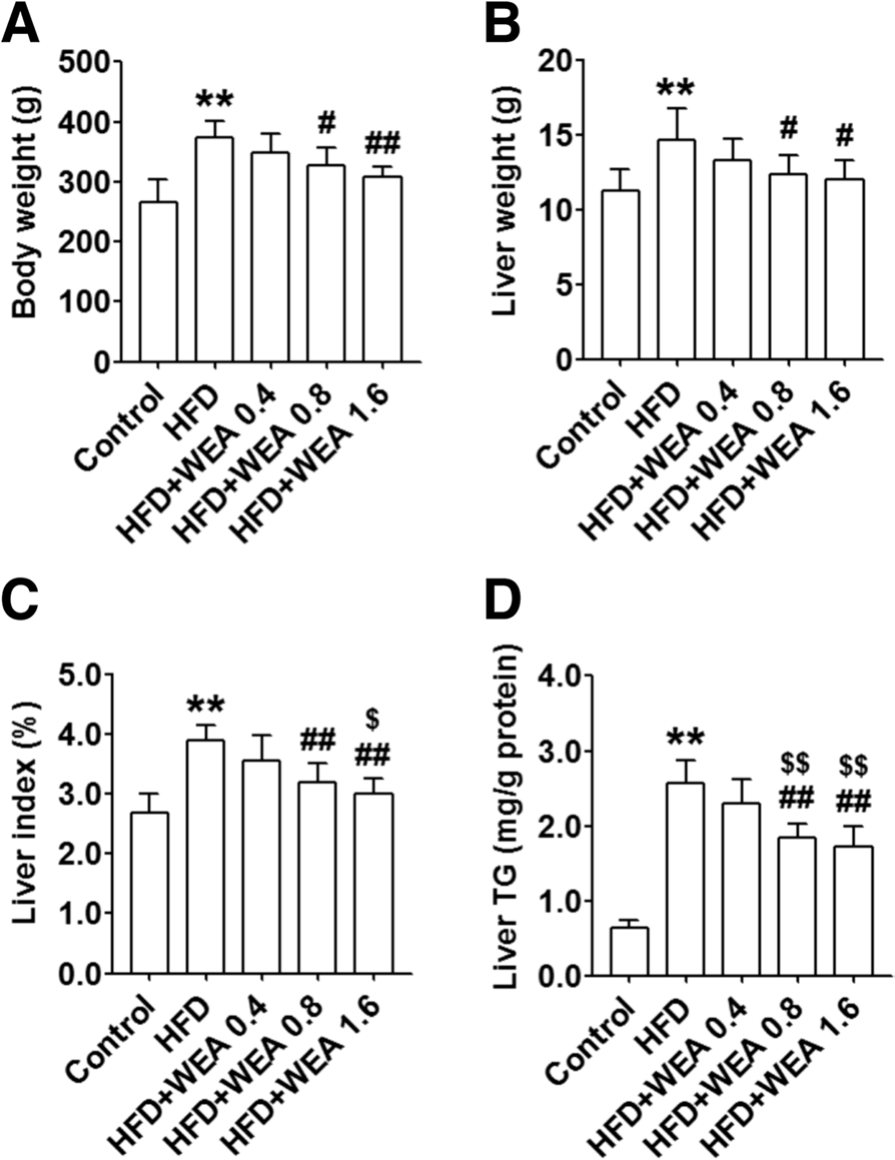
WEA decreases the body weight and liver steatosis of HFD-induced NAFLD rats. 8 weeks old male rats were fed HFD for 8 weeks, and then treatment with WEA at three dosage (0.4, 0.8, and 1.6 g/kg body weight) for 8 weeks. After the end of the intervention, the serum and liver tissues were collected. Body weight (**A**), liver weight (**B**), liver index (**C**), and liver TG content (**D**) were performed. The data are expressed as mean ± SD, *n* = 8 per group. ^**^P < 0.01 vs. Control group; ^#*P*^ < 0.05, ^##^*P* < 0.01 vs. HFD group; ^$^*P* < 0.05, ^$$^*P* < 0.01 vs. HFD + WEA 0.4 group

A significant increase in body weight (Fig. 2A) and liver weight (Fig. 2B) was observed in the HFD group compared with the control group (*P* < 0.01). Notably, the body and liver weights of animals in the HFD + WEA 0.8 and HFD + WEA 1.6 groups were lower than those in the HFD group (*P* < 0.05 and *P* < 0.01, respectively) (Figs. 2A–B). The liver index of the HFD group increased significantly (*P* < 0.01) compared to that of the control group, whereas treatment with WEA at 0.8 and 1.6 g/kg BW significantly decreased the liver index (*P* < 0.01) (Fig. 2C). Furthermore, the liver index was significantly reduced in the HFD + WEA 1.6 group (WEA 1.6 g/kg BW) compared to the HFD + WEA 0.4 group (WEA 0.4 g/kg BW) (*P* < 0.01) (Fig. 2C). Consistently, the TG content in the livers of animals in the HFD group was considerably increased compared to those in the control group (*P* < 0.01); however, liver TG content was significantly decreased in the HFD + WEA 0.8 and HFD + WEA 1.6 groups compared to the HFD and HFD + WEA 0.4 groups (*P* < 0.01) (Fig. 2D).

### Effect of WEA on serum lipid profiles and liver enzyme levels

**Fig. 3:**
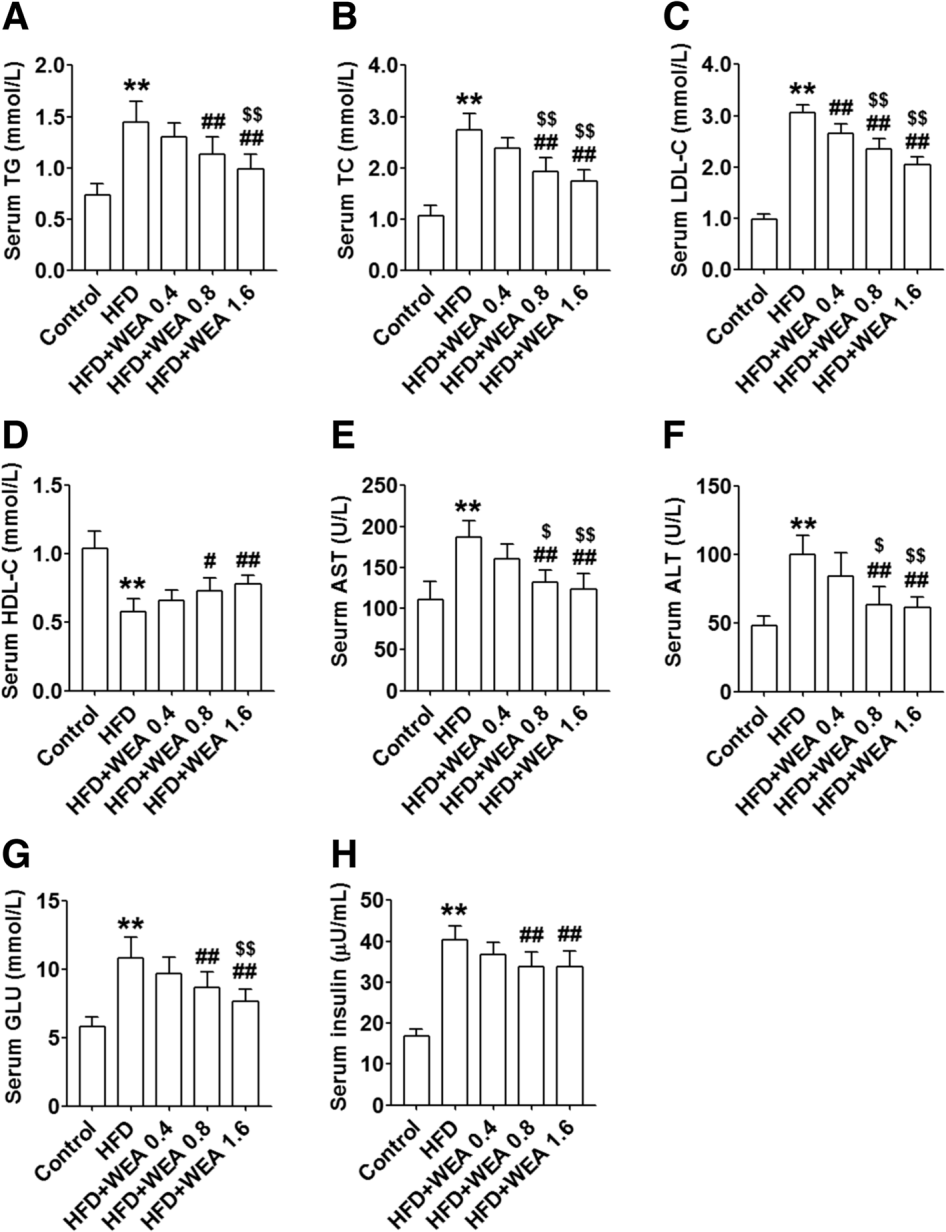
WEA improves lipid metabolism and liver functions in rats with NAFLD. The serum samples were collected from the rats in Fig. 2. Serum levels of TG (**A**), TC (**B**), LDL-C (**C**), HDL-cholesterol (**D**), AST (**E**), ALT (**F**), GLU (**G**), and insulin (**H**) were tested. The data are expressed as mean ± SD, *n* = 8 per group. ^**^P < 0.01 vs. Control group; ^#^*P* < 0.05, ^##^*P* < 0.01 vs. HFD group; ^$^*P* < 0.05, ^$$^*P* < 0.01 vs. HFD + WEA 0.4 group. TG: triglyceride; TC: total cholesterol; LDL-C: low density lipoprotein -cholesterol; HDL-C: high density lipoprotein-cholesterol; AST: aspartate transaminase; ALT: alanine aminotransferase; GLU: glucose

To evaluate the effect of WEA on metabolic homeostasis in rats with NAFLD, we measured serum biochemical parameters and changes in serum liver enzyme levels in these groups. As depicted in Fig. 3, there was a significant increase in the serum levels of TG, TC, LDL-C, AST, and ALT, and a decrease in serum HDL-C levels in the HFD group compared with those in the control group (*P* < 0.01). However, WEA at doses of 0.8 and 1.6 g/kg BW significantly decreased these serum biochemical parameters (*P* < 0.05) (Fig. 3) and increased serum HDL-C levels (*P* < 0.05) (Fig. 3D). Furthermore, the serum levels of TC, LDL-C, AST, and ALT were significantly reduced in the HFD + WEA 0.8 and HFD + WEA 1.6 groups (Fig. 3), while serum levels of TG were significantly reduced in the HFD + WEA 1.6 group, compared to the HFD + WEA 0.4 group (*P* < 0.05 or *P* < 0.01) (Fig. 3A). Together, these results suggest that WEA administration improves blood lipid metabolism and liver function in rats with HFD-induced NAFLD.

### Effect of WEA on hepatic oxidative stress

**Fig. 4:**
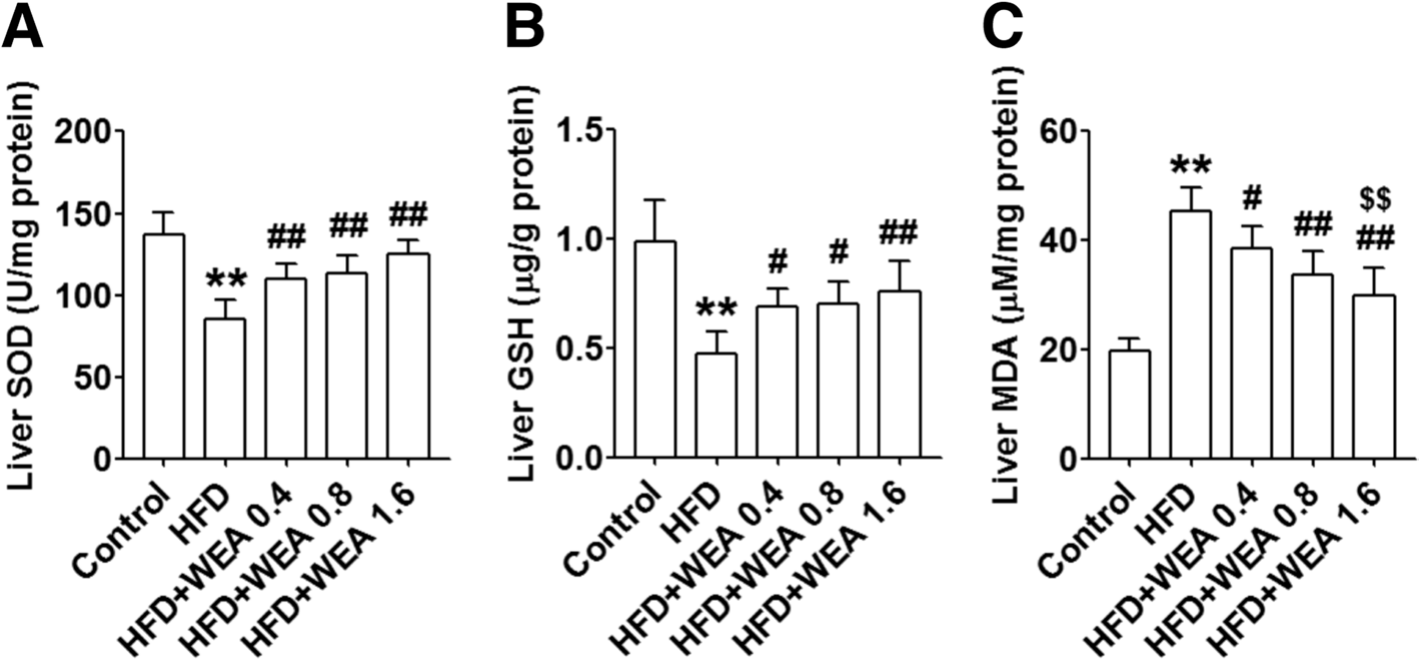
WEA increases hepatic antioxidant activity in NAFLD rats. The liver samples were collected from the rats in Fig. 2. Liver SOD activity (**A**), GSH content (**B**), and MDA content (**C**) were performed. The data are expressed as mean ± SD, *n* = 8 per group. ^**^P < 0.01 vs. Control group; ^#^*P* < 0.05, ^##^*P* < 0.01 vs. HFD group; ^$$^*P* < 0.01 vs. HFD + WEA 0.4 group. SOD: superoxide dismutase; GSH: glutathione; MDA: malondialdehyde

To evaluate the anti-oxidative stress effect of WEA in rats with NAFLD, we determined hepatic SOD activity as well as GSH and MDA levels in the investigated groups. We found that the activity of SOD and GSH in the livers of animals in the HFD group was significantly decreased (*P* < 0.01) compared with the control group (Fig. 4A and B). In contrast, the MDA content in the livers of animals in the HFD group was significantly higher (*P* < 0.01) than that of those in the control group (Fig. 4C). However, hepatic SOD activity and hepatic GSH were substantially increased in the WEA-treated groups compared with the HFD group (*P* < 0.05 and *P* < 0.01, respectively). In contrast, the hepatic MDA levels in the WEA-treated groups was significantly lower than that in the HFD group (*P* < 0.01), and the hepatic MDA levels in the HFD + WEA 1.6 group was much lower than that in the HFD + WEA 0.4 group (*P* < 0.01) (Fig. 4C).

### Effect of WEA on hepatic inflammatory regulation

**Fig. 5:**
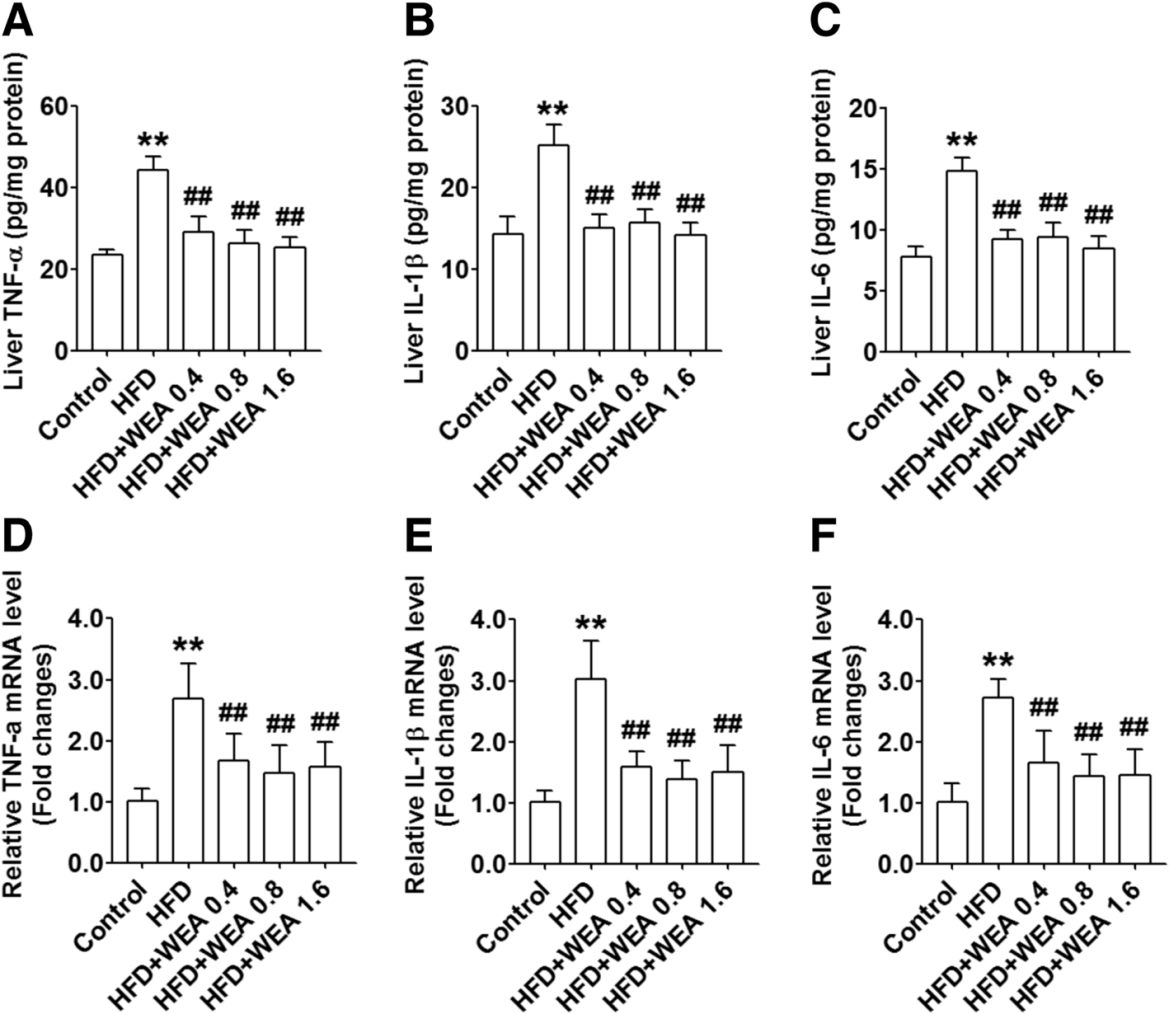
WEA reduces inflammation in the liver of NAFLD rats. The liver samples were collected from the rats in Fig. 2. Liver protein levels of TNF-α (**A**), IL-1β (**B**), and IL-6 (**C**) were performed with ELISA kits. The hepatic mRNA expression of TNF-α (**D**), IL-1β (**E**), and IL-6 (**F**) were tested using real-time PCR assay. The data are expressed as mean ± SD, *n* = 8 per group. ^**^P < 0.01 vs. Control group; ^##^*P* < 0.01 vs. HFD group. TNF-α: tumor necrosis factor alpha; IL-1β: interleukin-1 beta; IL-6: interleukin-6

To examine the anti-inflammatory activity of WEA in HFD-induced NAFLD rats, we measured the hepatic protein levels of pro-inflammatory cytokines (TNF-α, IL-1β, and IL-6) and the hepatic mRNA expression of these genes in HFD-fed rats after WEA administration. Hepatic protein levels of TNF-α, IL-1β, and IL-6 were higher in the HFD group than in the control group (*P* < 0.01) (Fig. 5A–C). However, the hepatic protein levels of these inflammatory cytokines were significantly reduced in animals in all the WEA-treated groups compared to those in the HFD group (*P* < 0.01). Consistently, WEA treatment significantly decreased the mRNA expression of these inflammatory genes (*P* < 0.01) (Fig. 5D–F).

### Effects of WEA on liver histopathology

**Fig. 6:**
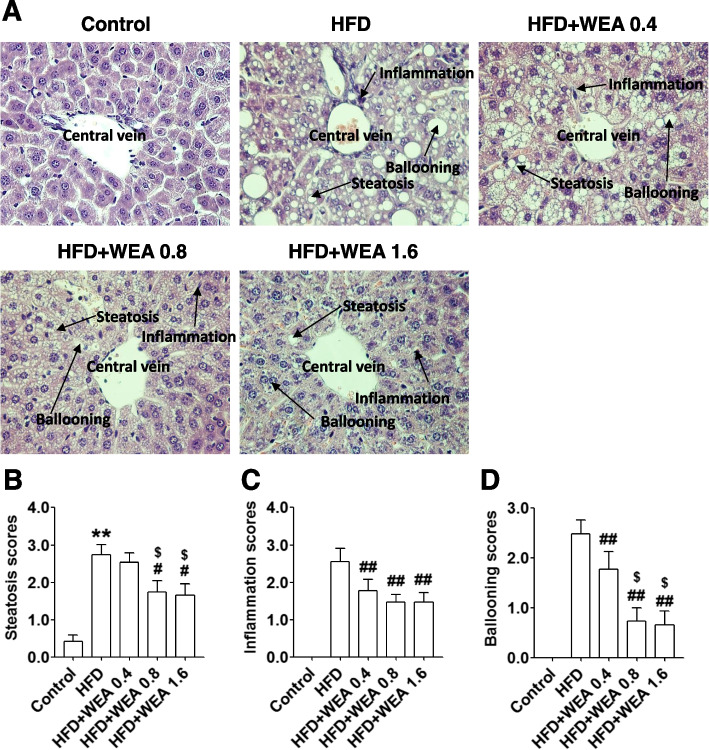
WEA attenuates liver injury in NAFLD rats. The liver section were prepared from the rats in Fig. 2, and histological assessments for liver injury in NAFLD rats were performed. **A** Representative images of hematoxylin and eosin (H&E)-stained sections of each group (magnification 400×). Quantification of the rats liver steatosis (**B**), lobular inflammation (**C**), and ballooning (**D**), respectively. The data are expressed as mean ± SD, *n* = 8 per group. ^**^P < 0.01 vs. Control group; ^#^*P* < 0.05, ^##^*P* < 0.01 vs. HFD group; ^$^*P* < 0.04 vs. HFD + WEA 0.4 group

As depicted in Fig. 6A, representative histopathological images of the liver from the control group shows normal architecture of the organ with healthy hepatocytes and the central vein. In contrast, the liver from the HFD group showed severe pathological changes, including steatosis, lobular inflammation, and ballooning. Rats with NAFLD treated with 0.4 g/kg BW WEA showed moderate fatty degeneration of hepatocytes and moderate parenchymatous infiltration of inflammatory cells in the liver; however, a considerable improvement in the histopathology of the livers was observed in rats from the HFD + WEA 0.8 and HFD + WEA 1.6 groups (Fig. 6A). Accordingly, quantification analysis demonstrated that the histological scores for steatosis, lobular inflammation, and ballooning in the livers of rats from the HFD + WEA 0.8 and HFD + WEA 1.6 groups were significantly lower than those in the HFD group (Fig. 6B–D). Furthermore, the histological scores for steatosis and ballooning in the HFD + WEA 1.6 group were much lower than those in the HFD + WEA 0.4 group (*P* < 0.05) (Fig. 6B, D).

### Effect of WEA on hepatic expression of lipid-metabolism genes

**Fig. 7:**
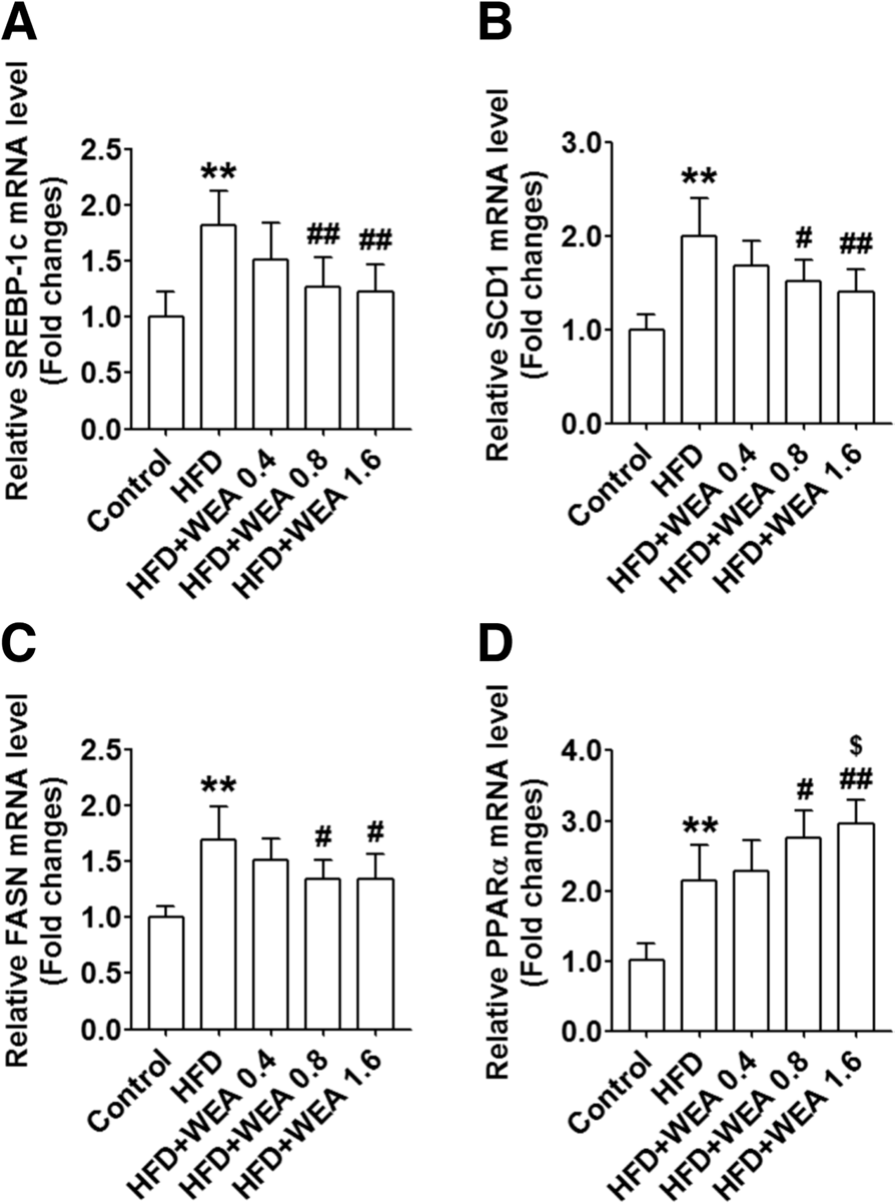
WEA improves hepatic lipid metabolic gene expression in NAFLD rats. The liver samples were collected from the rats in Fig. 2. The hepatic mRNA expression of SREBP1c (**A**), SCD1 (**B**), FASN (**C**), and PPARα (**D**) were tested using real-time PCR assay. The data are expressed as mean ± SD, *n* = 8 per group. ^**^P < 0.01 vs. Control group; ^#^*P* < 0.05, ^##^*P* < 0.01 vs. HFD group; ^$^*P* < 0.04 vs. HFD + WEA 0.4 group. SREBP1c: sterol regulatory element binding protein 1c; SCD1: stearoyl-coA desaturase 1; FASN: fatty acid synthase; PPARα: peroxisome proliferator-activated receptor alpha

To investigate the mechanisms underlying the role of WEA in the regulation of lipid metabolism, we evaluated the hepatic expression of genes involved in de novo lipogenesis and fatty acid oxidation. The expression of SREBP-1c, SCD1, FASN, and PPARα was significantly elevated in the HFD group compared to that in the control group (*P* < 0.05 or *P* < 0.01) (Fig. 7). However, there was a decrease in the expression of SREBP-1c, SCD1, and FASN, and an increase in the expression of PPARα in the HFD + WEA 0.8 and HFD + WEA 1.6 groups, compared with the HFD group (*P* < 0.05 or *P* < 0.01) (Fig. 7).

### Effect of WEA on liver insulin signaling

**Fig. 8:**
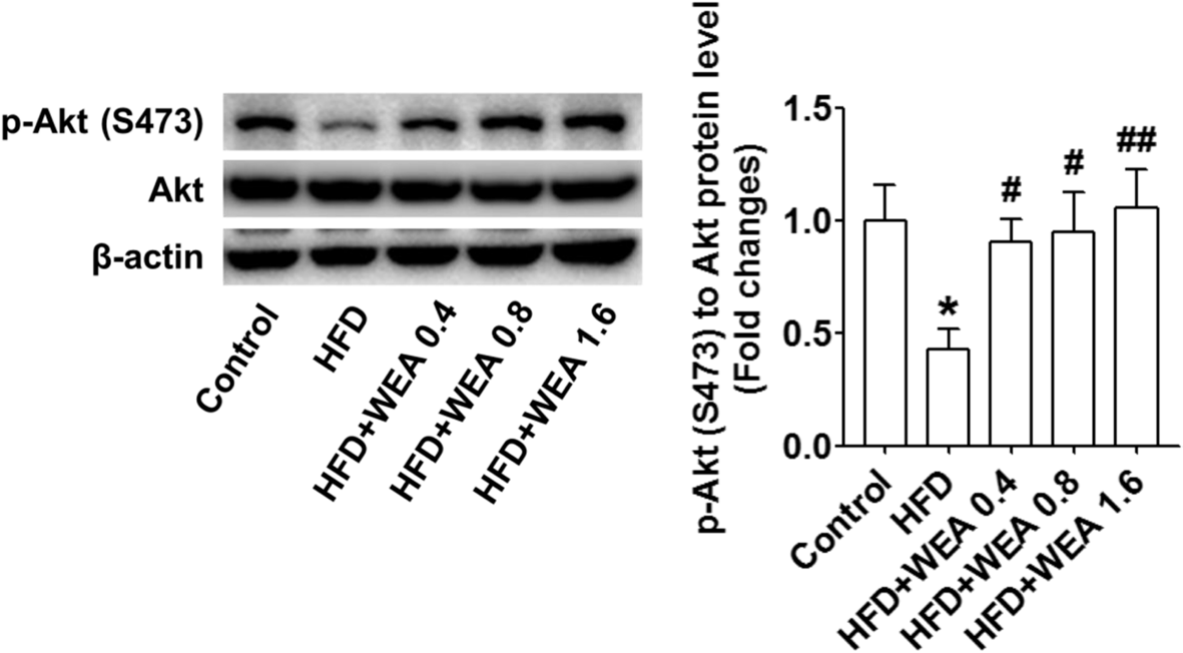
WEA increases insulin signaling in the liver of NAFLD rats. The liver samples were collected from the rats in Fig. 2. The protein levels of Akt and p-Akt (S473) in the liver of rats were tested using WB assay. The data are expressed as mean ± SD, *n* = 8 per group. ^*^P < 0.05 vs. Control group; ^#^*P* < 0.05, ^##^*P* < 0.01 vs. HFD group

To evaluate the role of WEA in liver insulin signaling, we measured the serum levels of GLU and insulin and evaluated the hepatic protein expressions of Akt and p-Akt (S473). The results revealed that serum GLU levels were significantly reduced in the HFD + WEA 0.8 and HFD + WEA 1.6 groups, compared with the HFD group (*P* < 0.01) (Fig. 3G), with these levels being much lower in the HFD + WEA 1.6 group than in the HFD + WEA 0.4 group (*P* < 0.01) (Fig. 3G). However, the variation in serum insulin levels was consistent with the change in serum GLU levels in the WEA-treated groups (Fig. 3H). As depicted in Fig. 8, the protein expression of Akt did not change between groups, whereas WEA treatment enhanced Akt phosphorylation at Ser473 in the livers of HFD-fed rats (Fig. 8).

## Discussion

Artichokes are widely recognized as herbal medicines because of their hepatoprotective effects [7]. In the present study, our findings showed that WEA administration (0.8 and 1.6 g/kg BW) reduced the serum levels of lipid profiles, increased hepatic antioxidant activity, improved hepatic inflammation, and ameliorated liver function in HFD-induced NAFLD rats. Histopathological analysis revealed that WEA treatment prevented liver injury in rats with NAFLD by reducing liver steatosis, lobular inflammation, and ballooning. WEA decreased the hepatic expression of lipogenic genes and increased the expression of fatty acid oxidation genes, which might contribute to the reduction of hepatic steatosis in HFD-induced NAFLD rats. Additionally, WEA may ameliorate liver insulin resistance by activating the PI3K/Akt signaling pathway.

A HFD has been widely used to create an animal model for NAFLD [19, 20]. Herein, our findings showed that the HFD-fed rats exhibited a significant increase in body weight and serum levels of TG, TC, LDL-C, ALT, and AST, and a decrease in serum levels of HDL-C, compared with control rats. Artichokes are well known for their lipid-lowering effects [21]. Accordingly, the results revealed that WEA administration at doses of 0.8 and 1.6 g/kg BW decreased serum lipid profiles, such as TG, TC, and LDL-C. HFD-induced lipid accumulation in the liver is modulated by both fatty acid uptake and de novo lipogenesis [22]. Our results showed that the decreased TG content in the livers of WEA-treated rats (0.8 and 1.6 g/kg BW) might be attributed to the reduced hepatic expression of SREBP-1c, SCD1, FASN genes, and increased expression of the PPARα gene, suggesting that WEA ameliorates hepatic steatosis by decreasing lipogenesis and increasing fatty acid oxidation in a HFD-induced NAFLD rat model. Elevated serum levels of liver enzymes such as ALT and AST is a well-recognized marker for liver injury and is also one of the diagnostic features of NAFLD [23]. Herein, WEA administration induced a significant decrease in serum levels of ALT and AST, which is consistent with previous reports [9, 24], implying that the hepatoprotective effects of WEA might be associated with decreased liver TG content.

Chlorogenic acid and cynarin are well known for their antioxidant activities [25, 26]. As mentioned above, the contents of chlorogenic acid and cynarin in WEA were 1.2 and 4.8%, respectively. As expected, our findings revealed that WEA administration at all three dosages exhibited antioxidant effects by decreasing hepatic MDA levels, increasing GSH concentration, and increasing SOD activity in the livers of rats with NAFLD, implying that the antioxidant activity of WEA might be attributable to the reduction in lipid peroxidation and augmentation of the endogenous antioxidant activity of the liver.

Our previous study demonstrated that ethanolic extracts from artichoke downregulated the expression of transcription factor NF-кb [15], which serves as a master regulator of inflammation and immune homeostasis and induces the gene expressions of a variety of pro-inflammatory cytokines (TNF-α, IL-1β, IL-6) upon stimulation [27]. In this study, our results revealed that WEA administration induced a significant decrease in hepatic TNF-α, IL-1β, and IL-6 levels, and the hepatic expression of TNF-α, IL-1β, and IL-6 genes was downregulated in WEA-treated rats with NAFLD, suggesting that the anti-inflammatory activity of WEA might be attributable to the inhibition of the hepatic expression of inflammation-related genes in this animal model. Together, histopathological analysis revealed that HFD-induced liver injury (including steatosis, lobular inflammation, and ballooning) was attenuated by WEA treatment at doses of 0.8 and 1.6 g/kg BW.

HFD-induced obesity is associated with insulin resistance and hyperglycemia [28]. Our findings showed that treatment with WEA (0.8 and 1.6 g/kg BW) induced a significant decrease in serum GLU and insulin levels, suggesting that reduction of serum GLU might not possibly be related to the reduced serum insulin levels in WEA-treated rats with NAFLD. The PI3K-Akt signaling pathway plays a potential role in the insulin signaling pathway, which is considered a key regulator of gluconeogenesis and glycogen synthesis [29]. Western blot analysis revealed that WEA administration reduced liver insulin resistance by increasing Akt phosphorylation at Ser473. Similarly, one study demonstrated that artichokes reduced postprandial glycemic and insulinemic responses in normal subjects [7]. Because inflammation promotes the development of insulin resistance in tissues such as the liver, skeletal muscle, and adipose tissue by inhibiting insulin signal transduction [30], we propose that the improved insulin signaling in the liver might partly be attributable to the reduced inflammation observed in WEA-treated rats with NAFLD.

## Conclusions

Our findings revealed that WEA administration successfully mitigated steatosis, oxidative stress, inflammation, and insulin resistance in the livers of rats with NAFLD induced by HFD, indicating that WEA could combat NAFLD by efficiently targeting the “multiple-hits,” Overall, these findings provide a proof-of-concept for the protective role of WEA against NAFLD.

## Supplementary Information

**Additional file 1.**

## Data Availability

All data generated or analysed during this study are included in this published article.
