# Supplementary figures and images for: Water extract from artichoke ameliorates high-fat diet-induced non-alcoholic fatty liver disease in rats
Source: BMC Complement Med Ther. 2022 Nov 24;22:308. doi: 10.1186/s12906-022-03794-9 (PMC9686119; doi:10.1186/s12906-022-03794-9)

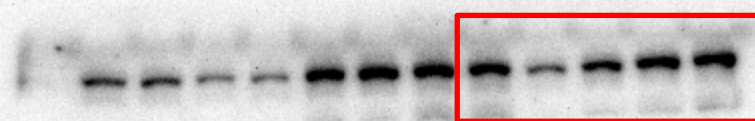

**p-Akt(S473)**

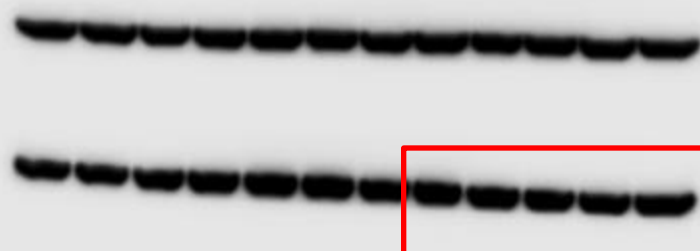

**Akt**

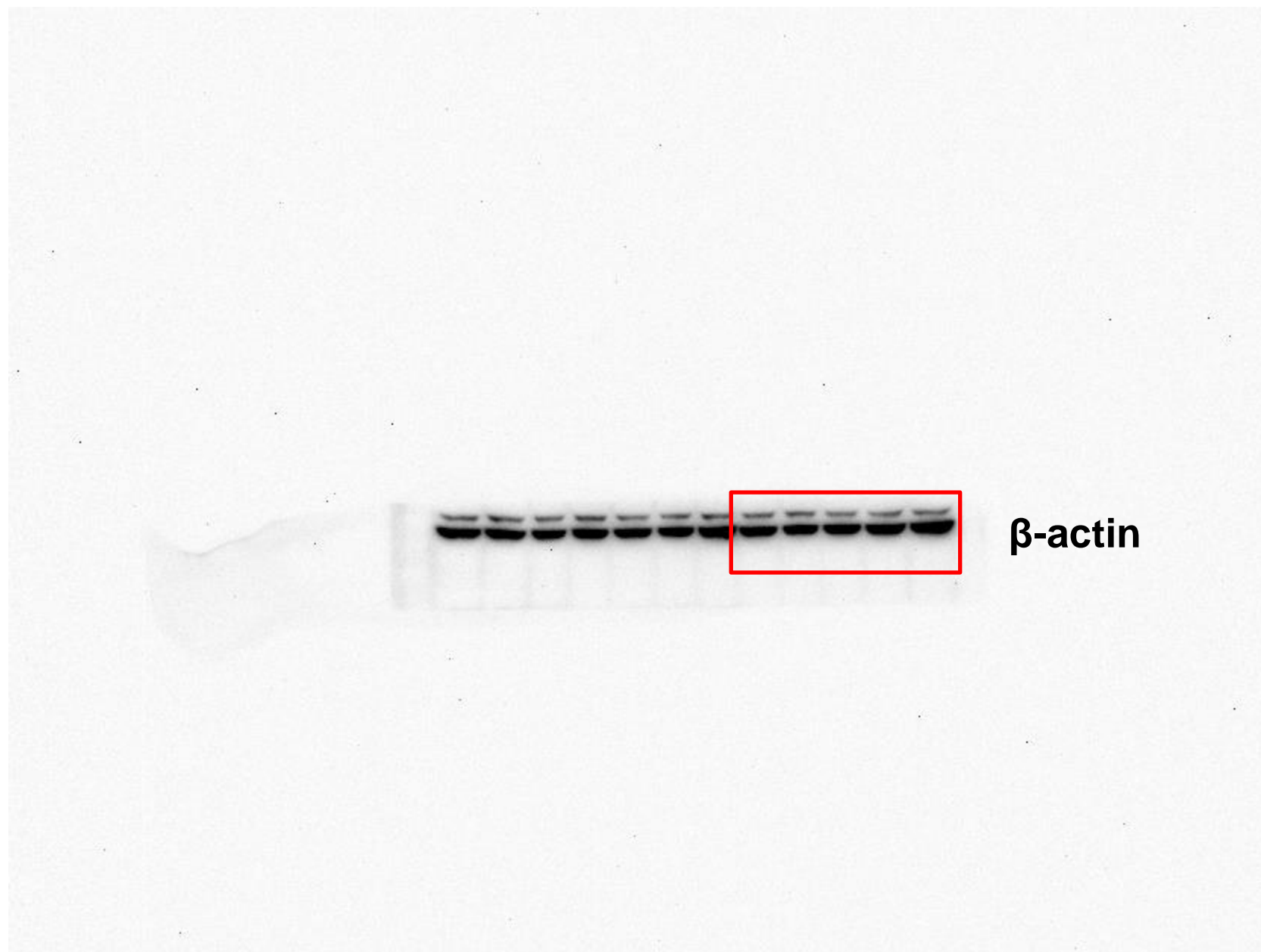

**p-Akt (S473)**

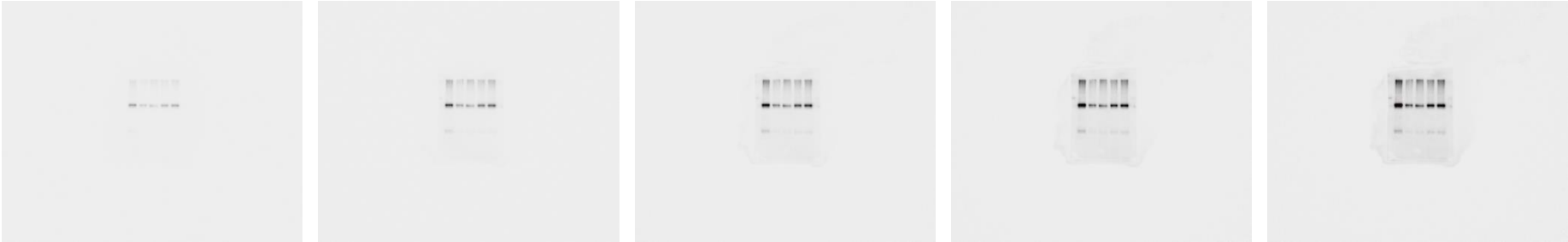

**Akt**

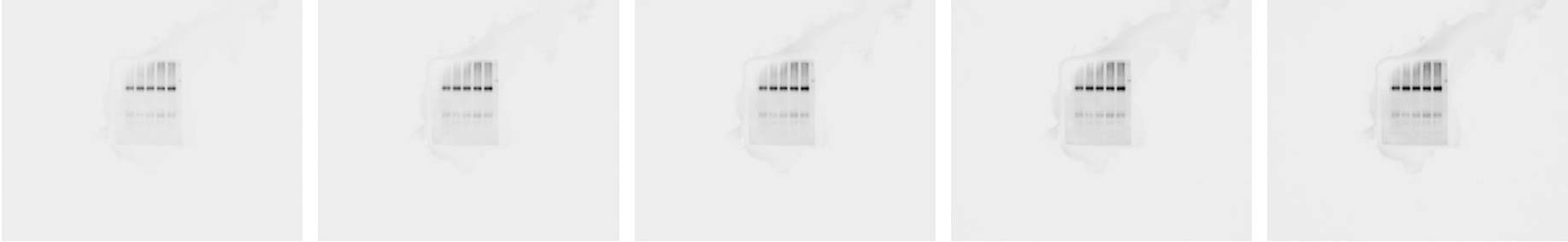

**p-Akt (S473)**

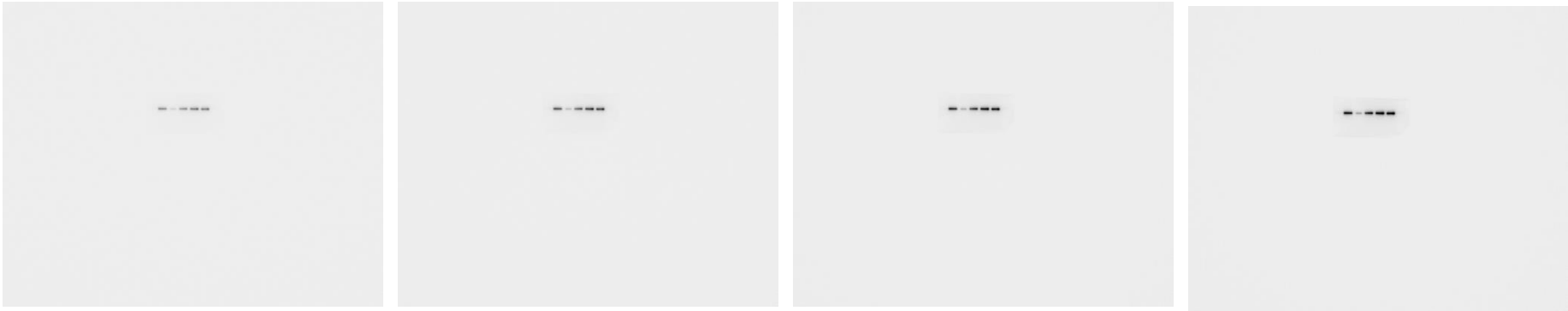

**Akt**

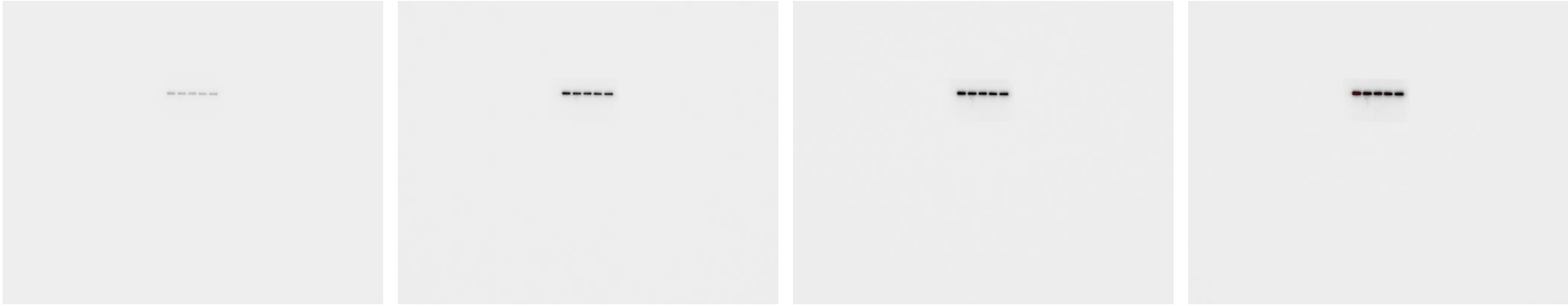

Supplement: Supplementary file 1 — Additional file 1. [file 12906_2022_3794_MOESM1_ESM.pdf]
